# COVID-19 restrictive control measures and maternal, sexual and reproductive health issues: risk of a double tragedy for women in sub-Saharan Africa

**DOI:** 10.11604/pamj.2021.40.122.27946

**Published:** 2021-10-28

**Authors:** Grant Murewanhema, Mugove Gerald Madziyire

**Affiliations:** 1 Department of Obstetrics and Gynaecology, University of Zimbabwe College of Health Sciences, Harare, Zimbabwe,; 2 Society of Obstetricians and Gynaecologists (ZSOG), Harare, Zimbambwe

**Keywords:** COVID-19, pandemic, lockdowns, maternal, sexual and reproductive health, sub-Saharan Africa

## Abstract

Governments in sub-Saharan Africa implemented restrictive measures, including lockdowns, to curb the spread of COVID-19, without measures to protect women and girls. Evidence from previous humanitarian crises in resource-limited settings in sub-Saharan Africa indicates that these populations may suffer disproportionately from the effects of the restrictive control measures, owing to differential access to services, including maternal, sexual and reproductive health services. These services are time-sensitive, and delays and disruptions introduced by the restrictive measures may result in adverse consequences, including increased maternal and perinatal morbidity and mortality. Therefore, governments must find ways of ensuring continuity of these essential services during pandemic times, in a conducive environment, protective to both care providers and care seekers. Surveillance of the impact of the pandemic must be ongoing to inform practice and refine public health interventions, as the indirect effects of the COVID-19 pandemic might be worse than the direct effects.

## Perspective

The SARS-CoV-2 had infected approximately 231,416,660 individuals and claimed almost 4,741,330 lives as reported to the World Health Organisation (WHO) by 26^th^ September 2021 [1,2]. Africa had 5,998,863 confirmed cases with 144,957 cumulative fatalities [2]. The aggressive virus continues to infect substantial proportions of the world´s population, despite the introduction of vaccination programmes globally [3,4]. As new SARS-CoV-2 variants of concern continue to emerge, and further waves of COVID-19 arise, countries continue to impose restrictive measures on their populations, including lockdowns, to break chains of transmissions [5]. In some countries such as those in sub-Saharan Africa, lockdowns were not accompanied by measures to protect women and girls [6]. Evidence from previous humanitarian crises in Africa, such as the Ebola viral disease outbreaks in West Africa has showed that women from such settings may be affected disproportionately by the pandemic, directly and indirectly. Differential access to needs exposes them, during and beyond the pandemic. A maternal, sexual and reproductive health (MSRH) framework, emphasizing human rights, acknowledging intersecting injustices and recognizing power structures, is essential for addressing and monitoring the inequitable gender, health and social effects of the pandemic [6]. In the resource-limited settings of sub-Saharan Africa, indirect effects can be more severe than the pandemic itself [7]. This is especially so as the direct magnitude of COVID-19 in Africa has not been as devastating as initially projected. The indirect effects come in the form of movement restrictions, lost income, stock-outs due to supply chain disruptions and cessation of non-emergency medical care. Movement restrictions reduce timeous access to services as people are delayed by limitation in public transport and the need to justify movements. Loss of income through reduced economic activity reduces access to essential MSRH care. Many people in developing countries experience poor communication emanating from poor roads, digital networks and public transport systems. Pre-existing challenges in surveillance in sub-Saharan Africa complicate accurate assessment of the direct and indirect effects of the COVID-19 pandemic on healthcare provision. We describe the effects of the COVID-19 pandemic and resultant restrictive control measures such as lockdowns on provision of MSRH services for women in sub-Saharan Africa. Effects on maternal, sexual and reproductive health services provision for women in sub-Saharan Africa.

The lockdown restrictions have resulted in markedly reduced provision of care for women seeking MSRH services in resource-limited settings. Hospitals switched to emergency mode to avert the potential spread of COVID-19. Despite substantial progress in knowledge and control of COVID-19, including introduction of vaccination programmes, countries such as Zimbabwe have continued to offer limited healthcare services, including MSRH services, with reduced attendances in antenatal care clinics, outpatient gynaecology clinics, family planning and other sexual and reproductive health services. News of healthcare workers (HCWs) dying from COVID-19 brought anxiety among health service providers [8,9]. The exodus of essential healthcare from sub-Saharan Africa to countries such as the United Kingdom, the United States of America and Australia has reportedly worsened due to gaps created in the West by the COVID-19 pandemic, and has worsened healthcare shortages in Zimbabwe and other African countries. For women, this may result in aggravation of complications from delayed obstetric, gynaecologic and sexual and reproductive health care. In maternity, potential complications are usually averted before they occur, and obstetric conditions are time-sensitive, with delays in interventions often leading to catastrophic consequences with increased maternal and perinatal morbidity and mortality. Estimates show that a reduction of 10% coverage in contraceptive services could add 15 million unintended pregnancies in 1 year, an additional 28,000 maternal deaths and 168,000 new-born deaths [10,11]. Likewise, a 10% reduction in access to safe abortions in 1 year could increase unsafe terminations by 3.3 million resulting in an additional one thousand maternal deaths. Reduction in maternal health coverage of 10-50% could result in an increase of 60% to maternal deaths [11]. In Sierra Leone, Ebola-related direct mortality ended up almost equivalent to indirect mortality due to disruption of maternal and new-born care [12]. Patients with high-risk pregnancies in these resource-challenged settings may end up not getting adequate antenatal surveillance. These high-risk pregnancies include patients with previous caesarean sections, hypertensive disorders of pregnancy, multiple gestations and chronic medical conditions, among others. Some present for the first time with complications such as ruptured uteri and eclampsia. Unpublished comparative audits at two hospitals in Zimbabwe showed a reduction in total deliveries and a trend towards rise in maternal and perinatal mortality in March-May 2020 compared to the same period in 2019 [13]. Many are unbooked, have no obstetric scans and HIV test results, and are not receiving micronutrient supplements.

Those requiring elective surgery are presenting in labour, which can affect maternal and foetal outcomes. Support services including blood donations are negatively impacted by the restrictive measures, with blood donors in some African countries mainly being schoolchildren. With COVID-19 related school closures, there was a shortage of donors. Fortunately, the situation is improving with the gradual re-opening of educational institutions. Moreover, some countries such as Zimbabwe import reagents for processing blood and are affected by supply chain disruptions, resulting in blood product shortages [14]. Blood products are often life-saving for fatal obstetric and gynaecologic conditions such as obstetric haemorrhage, ruptured ectopic pregnancies and disseminated intravascular coagulation, if they are administered on time before irreversible complications set in. Indirect causes of maternal mortality such as HIV and malaria are affected through closure of routine care, re-allocation of resources and supply chain disruptions. HIV among women of reproductive age is still highly prevalent in sub-Saharan Africa, with United Nations Programme on HIV/AIDS (UNAIDS) estimating that close to 60% of incident HIV infections are still occurring in this population, which remains a key population for epidemic control. It is estimated that an interruption of highly active antiretroviral therapy (HAART) over 6 months could result in an excess of 500,000 deaths in sub-Saharan Africa in 4 years and double vertical transmission of HIV [15]. HIV pre-exposure and post-exposure prophylaxis services could also be disrupted, with victims of sexual assault and gender-based violence, which have been widely reported as increased, failing to access these services, as well as prophylaxis for sexually transmitted infections and emergency contraception. Reports of reduced access to HIV testing and counselling services, which act as gateways to HIV care and treatment have also been circulated. Interruption in intermittent presumptive treatment could cause an upsurge of malaria in pregnancy, which is fatal, and accounts for a sizeable number of maternal deaths annually in some sub-Saharan Africa regions. Overall, there could be an increase in maternal mortality from indirect causes in a region which still has some of the highest maternal mortality rates globally, and struggling to attain the third sustainable development goal (SDG).

Sexual activities persist even in pandemics, and might reportedly even increase in some resource-limited settings with increased idleness. Hence, measures to ensure access to contraception and early pregnancy care are necessary to minimise unintended pregnancies, unsafe terminations, septic miscarriages and consequent maternal mortality. Experience from previous crises showed reduced access to these essential services, with undesirable consequences, especially for adolescent girls and young women [7-12]. Adolescent girls constitute a special population, whose sexual and reproductive health needs are overlooked in sub-Saharan Africa, more so during crisis times. The focus on most sub-Saharan African countries, especially during the early phases of the pandemic, was on controlling the spread of the virus, shifting attention from other issues of public health importance such as the provision of MSRH services for women. Poorly planned policies result in increased maternal and infant mortality, sexually transmitted infections, depression, suicide and intimate partner violence, exchanging direct COVID-19 related mortality with indirect COVID-19 related morbidity and mortality due to neglect. Systemic racism, xenophobia and discrimination, which are rife in some African countries, may further compound logistical barriers to accessing MSRH care for women. Untreated sexually transmitted infections can also have long-term consequences for a woman´s reproductive health including subfertility, ectopic pregnancies and chronic pelvic pain; therefore, failing to access timely essential MSRH services not only has short term consequences, but can also have long term biological and psychosocial consequences on a woman´s life.

**Recommendations:** with the advent of SARS-CoV-2 vaccination, it has become important to ensure that all willing and eligible frontline healthcare workers providing essential healthcare services such as MSRH services are prioritized. Vaccination protects the healthcare workers and their clients from the spread and adverse outcomes associated with COVID-19. The increased availability of vaccines in sub-Saharan Africa will gradually improve the provision of clinical services to women and girls, and the return of most aspects of life to normalcy. However, before countries in sub-Saharan Africa reach their herd immunity thresholds, and even beyond, standard infection prevention and control (IPC) measures for curbing the spread of SARS-CoV-2 must continue to be observed. Action-oriented approaches to ensuring continued access to services during times of restrictive measures including lockdowns are required. These can be designed from guidance for ensuring continuity of essential health services during the COVID-19 pandemic from the WHO [10]. This encompasses prioritizing essential health services, optimizing service delivery settings, establishing safe and effective patient flow at all levels of care and rapidly optimizing workforce capacity. Additionally, they advise maintenance of the availability of essential medicines, equipment and supplies, adequate funding for public health and removing financial barriers to access. Specific to maternal and newborn health, the WHO recommends maintenance of antenatal and postnatal care, and essential auxiliary services, including laboratories and blood banks [10]. Unattended childbirth is more fatal than the risk of COVID-19 transmission in facilities where adequate IPC measures exist. Maintenance of maternal waiting shelters where logistical barriers to accessing healthcare facilities occur is recommended. Provision of long acting reversible contraception to reduce health facility visit frequency is ideal. We recommend up to three months supplies of oral contraceptives, micronutrients and antiretroviral medicines to minimize exposure to potentially infectious environments. Telemedicine can be effective in managing maternity patients while limiting exposure of personnel and patients to infection [16]. This is supported in a review by DeNicola [17]. Free or subsidized internet access for tele-consultation must therefore be considered. HCWs must be provided with adequate personal protective equipment (PPE) [10]. Important is continued education on IPC, including using PPE. The WHO offers guidance on adequate PPE for certain tasks [18]. There are reports of personnel who contracted COVID-19 despite adequate PPE because they could not use it properly. Offering competitive remuneration to maintain motivation is critical as HCWs practice in a hazardous environment. Access to MSRH services including maternal and newborn health, contraception, post-abortion care, sexual and menstrual health, cervical cancer screening and gender-based violence care is essential. Protocols to ensure their continuity are required. Authorities must allow women to travel to access these services. Utilization of pre-existing structures, including village health workers to distribute contraceptives, micronutrient supplements and anti-malarial medicines is worth considering. There is need to provide MSRH services in quarantine centers where cases have been clustered in some countries such as Zimbabwe. Ensuring humane conditions for women and girls in these centers is critical. Experienced, empathetic practitioners are needed to offer services to the needy. Women must continue to access contraception to avoid unintended pregnancies. Those pregnant in quarantine must be reviewed appropriately to optimize maternal and perinatal outcomes. Summarized recommendations are provided in Table 1.

**Table 1:** summarised recommendations for providing and maintaining essential maternal, sexual and reproductive health services during the COVID-19 pandemic

Item	Action
Antenatal and postnatal care	Where non-existent, initiate client booking systems to limit numbers
	Ensure adequate room for physical distancing
	Provide adequate PPE to healthcare workers
	Put in place triage systems for clients according to risk to ensure high-risk pregnancies are prioritised
	Appropriate health education to clients
Labour and delivery	Ensure adequate IPC measures in labour and delivery units
	Limit numbers of visitors to maternity units
	Provide adequate PPE
	Restore elective surgeries
Family planning, including provision of emergency contraception	Restore family planning services with adequate IPC measures
	Provide long-acting methods of contraception to willing patients
	Put in place mechanisms to ensure access to emergency contraception, especially to victims of rape
	If possible, restore rape clinics
HIV testing, counselling, care and treatment services and screening and treatment for sexually transmitted infections	Ensure three to six months supply of antiretroviral drugs for all clients on antiretroviral therapy
	Mechanisms to allow telemdicine consultations for minor ailments for these patientsPrioritise restoration and maintenance of HIV testing and counselling services
	Use community art refill groups for antiretroviral drug supplies
	Ensure timely access and testing for STIs to avert potential short and long term complications
Sexual and gender-based violence	Ensure timely access to essential services for these victims

## Conclusion

Evidence from previous humanitarian crises shows that MSRH outcomes for women and girls in resource-limited settings such as those in sub-Saharan Africa are neglected. Women are disproportionately affected by such, with undesirable maternal and perinatal outcomes, including increased maternal morbidity and mortality. Emerging evidence during the COVID-19 pandemic is also showing the same, raising the need for strong surveillance systems, especially in low resource settings, to monitor the direct and indirect impacts of the pandemic. Governments and other relevant stakeholders in MSRH in sub-Saharan Africa must prioritize development of policies and practices that protect women from the impacts of the pandemic. Regular audits to detect trends in MSRH are necessary to inform ongoing mitigation efforts.
